# Morphology-Driven SERS Activation in TMDCs: A Dual-Mode Platform for Sensorics and Theranostics

**DOI:** 10.3390/nano16090546

**Published:** 2026-04-30

**Authors:** Nadezhda M. Belozerova, Andrei A. Ushkov, Dmitriy V. Dyubo, Alexander V. Syuy, Alexander I. Chernov, Andrey A. Vyshnevyy, Sergey M. Novikov, Gleb I. Tselikov, Aleksey V. Arsenin, Vladimir G. Leiman, Valentin S. Volkov

**Affiliations:** 1 Moscow Center for Advanced Studies, Kulakova Str. 20, Moscow 123592, Russia; nmbelozerova@jinr.ru (N.M.B.); ushkov.andrei.a@gmail.com (A.A.U.); dmitriydyubo@gmail.com (D.V.D.); alsyuy@xpanceo.com (A.V.S.); vyshnevyy@xpanceo.com (A.A.V.); serjikn@gmail.com (S.M.N.); arsenin@xpanceo.com (A.V.A.); leimanvg@gmail.com (V.G.L.); 2 Frank Laboratory of Neutron Physics, Joint Institute for Nuclear Research, Joliot-Curie Str. 6, Dubna 141980, Russia; 3 Emerging Technologies Research Center, XPANCEO, Internet City, Emmay Tower, Dubai P.O. Box 393047, United Arab Emirates; celikov@xpanceo.com; 4 Department of General Physics, Perm National Research Polytechnic University, Perm 614990, Russia

**Keywords:** van der Waals materials, tungsten diselenide, tungsten ditelluride, laser ablation, ultrasonic exfoliation, nanoparticles, SERS, photoheating

## Abstract

The development of reproducible and stable plasmon-free substrates for surface-enhanced Raman scattering (SERS) is critical for practical applications in analytical chemistry. Transition metal dichalcogenides (TMDCs) have emerged as promising candidates due to their unique electronic properties, yet their performance is often constrained by the chemical inertness of their pristine basal planes. This work presents a systematic comparison of crystalline flakes and nanoparticles of tungsten diselenide (WSe_2_) and tungsten ditelluride (WTe_2_), prepared via liquid-phase ultrasonic exfoliation and non-equilibrium femtosecond pulsed laser ablation in liquid (PLAL), respectively. The results demonstrate that nanoparticle-based substrates consistently outperform their flake-based counterparts, achieving enhancement factors in the range of $10^{4}$. The superior performance of the nanoparticles is hypothesized to originate from the synthesis-induced defects and high-curvature regions in the nanoparticles shell which facilitates efficient, defect-mediated charge transfer between the substrate and the analyte. At the same time, the inner polycrystalline volume conserves the important characteristics of the bulk counterparts like excitons in semiconducting WSe_2_ and broadband absorption in semimetallic WTe_2_, which unblocks the tunable photothermal colloidal response. The study establishes morphology engineering through non-equilibrium synthesis as a powerful and generalizable strategy for designing high-performance, dual-function colloidal platforms, offering a pathway toward robust and reproducible analytical systems.

## 1. Introduction

Ultrasensitive and reproducible molecular detection is a primary source of innovations across multiple fields from medical diagnostics and environmental monitoring to national security [1,2,3,4,5]. Surface-enhanced Raman scattering (SERS) is a powerful analytical technique, capable of identifying molecules through their unique vibrational fingerprints with sensitivities extending to the single-molecule level [6,7,8,9].

Traditionally, SERS performance was improved through an electromagnetic mechanism, where localized surface plasmon resonances (LSPRs) in noble metals nanostructures generate intense electromagnetic “hot spots” [10,11,12]. These hot spots can amplify Raman signals by factors of $10^{6}$ to $10^{14}$, enabling extraordinary detection limits [1,13,14]. However, the plasmon-based SERS faces fundamental challenges that block its widespread practical implementation. First, the stochastic and often uncontrollable formation of hot spots leads to significant signal variations from sample to sample and complicates the quantitative analysis [2,10,15]. Further drawbacks, including the chemical instability of plasmonic materials (e.g., the oxidation of silver), potential cytotoxicity, and undesirable catalytic activity that can degrade the analyte, have motivated a search for alternative materials [15,16,17].

Plasmon-free SERS substrates represent a family of alternative materials, where signal enhancement is governed predominantly by a chemical mechanism (CM) [9,18,19]. The CM originates from a photoinduced charge transfer (PICT) process between the substrate and an adsorbed analyte molecule [12,14,20]. This charge transfer transiently alters the molecule’s polarizability, thereby increasing its Raman scattering cross-section [21].

Two-dimensional (2D) materials, in particular transition metal dichalcogenides (TMDCs), are a promising platform for exploring CM-SERS [9,20,22,23] due to their tunable electronic properties, exceptional chemical stability, and biocompatibility [5,20,24]. Despite the benefits, a high-performance SERS on the layered TMDCs suffers from chemically inert basal planes [25,26,27] with coordinatively saturated surface atoms, which severely limits the number of active sites available for the strong chemisorption and efficient charge transfer [28]. To unlock the full potential of these materials, a deliberate strategy of structural perturbation, commonly known as defect engineering, is required to activate the passive surface [9,29]. These defect states (i.e., chalcogen vacancies as point defects and TMDC layers edges as line defects) can function as mediators for charge transfer, enabling a resonant PICT process [18,19,21,25,28,30].

Recent advances have shown that TMDC-based SERS can be dramatically amplified through phase engineering, alloy engineering, and mixed-dimensional charge-transfer heterostructures. At the same time, many of the highest-performing platforms rely on monolayer films, heterostructures, or metal-templated architectures optimized specifically for ultimate charge-transfer resonance [31,32,33,34]. In contrast, colloidally processable, fully plasmon-free TMDC nanomaterials that simultaneously provide reproducible SERS activity and photothermal functionality remain comparatively underexplored. We, therefore, deliberately selected WSe_2_ and WTe_2_ as a tungsten-based TMDC pair with complementary electronic structures—exciton-dominated semiconducting WSe_2_ and semimetallic WTe_2_—to isolate how band structure influences charge-transfer-mediated SERS and NIR photothermal behavior while minimizing variation in the transition-metal chemistry.

In this work, a femtosecond pulsed laser ablation in liquid (PLAL) is used as a one-shot technique for TMDC nanostructuring and defect engineering. In contrast to conventional nanostructuring methods (i.e., liquid-phase ultrasonic exfoliation), which are quasi-equilibrium processes largely preserving the low-defect structure of the parent material [30,35,36,37,38], PLAL is an essentially non-equilibrium technique [39,40,41]. The ultrashort laser pulses generate TMDC plasma plumes with extreme temperatures and pressures; the subsequent ultrafast quenching kinetically traps the material in a high-energy, defect-rich state, promoting the formation of point surface defects even while maintaining overall crystallinity [42].

Within this study, we demonstrate that the non-equilibrium PLAL synthesis realizes the defect engineering in TMDC nanoparticles, which leads to their 10-fold increase in CM-SERS performance in comparison with flake-like analogues produced via exfoliation [43,44,45]. To validate this, a direct and systematic comparison of the SERS activity of crystalline flakes and nanoparticles of WSe_2_ and WTe_2_ is conducted. Interestingly, we reveal that the introduced defects do not alter significantly the nanoparticle volumetric optical response, allowing the use of bulk crystal optical constants for optical simulations. Specifically, it was successfully used for validating the experimentally observed photothermal conversion efficiency of WSe_2_ (above 40%) and WTe_2_ (above 80%) NPs. This investigation aims to establish morphology engineering as a robust and generalizable strategy for designing the next generation of high-performance sensing and theranostic platforms.

## 2. Materials and Methods

### 2.1. Synthesis of TMDC Nanomaterials

Bulk crystals of WSe_2_ and WTe_2_ were used as the precursor materials for the synthesis of both nanoparticles and flakes.

#### 2.1.1. Nanoparticle Synthesis via Femtosecond PLAL

**Figure 1:**
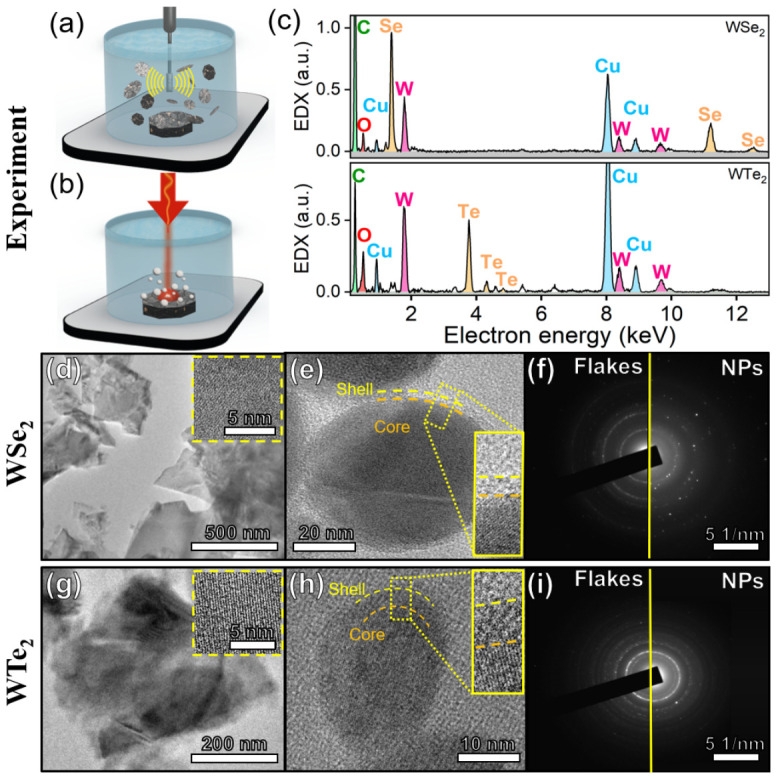
(**a**,**b**) Schematic image of ultrasonic exfoliation (**a**) and femtosecond pulsed laser ablation in liquid (PLAL) (**b**); (**c**) Energy-dispersive X-ray spectroscopy (EDX) characterization of PLAL-synthesized WSe_2_ and WTe_2_ nanoparticles (NPs); (**d**–**f**) WSe_2_: transmission electron microscopy (TEM) photograph of (**d**) flakes and (**e**) ablated NPs with zoomed areas in the insets; (**f**) Selected area electron diffraction (SAED) characterization of flakes and PLAL-synthesized WSe_2_ NPs; (**g**–**i**) WTe_2_: TEM photograph of (**g**) flakes and (**h**) ablated NPs with zoomed areas in the insets; (**i**) SAED characterization of flakes and PLAL-synthesized WTe_2_ NPs.

Nanoparticles were synthesized using femtosecond pulsed laser ablation in liquid (PLAL) (Figure 1a). A Yb:KGW femtosecond laser system (TETA-10, Avesta, Moscow, Russia) was employed, generating pulses with a wavelength of 1030 nm, a duration of 270 fs, a repetition rate of 1 kHz, and a pulse energy of 100 µJ. The bulk TMDC crystal was placed at the bottom of a glass cuvette filled with 2 mL of deionized water (DI water). The laser beam was focused by a 100 mm focal length lens, creating a spot with a diameter of approximately 50 µm on the target surface, corresponding to an energy density of about 5 J/cm^2^. To ensure uniform ablation, the cuvette was scanned over a 2 × 2 mm area at a speed of 5 mm/s using motorized translation stages. The total irradiation time for each synthesis was 15 min.

#### 2.1.2. Flake Synthesis via Ultrasonic Exfoliation

Flakes of the TMDC materials were prepared by liquid-phase ultrasonic exfoliation (Figure 1b) [46]. A probe-tip ultrasonic processor (CL-18, Qsonica L.L.C., Newtown, CT, USA) delivered 100 W of ultrasonic power for 4 h to a suspension containing fragments of the bulk crystal. The initial concentration of the TMDC material in the deionized water was 2 mg/mL.

### 2.2. Structural and Morphological Characterization

The structure and morphology of the resulting colloidal solutions of nanoparticles and flakes were analyzed using transmission electron microscopy (TEM), high-resolution TEM (HRTEM), and selected area electron diffraction (SAED). These investigations were performed on a JEOL JEM 2010 system (JEOL Ltd., Akishima, Tokyo, Japan) operated at an accelerating voltage of 200 kV. The JEOL JEM-2100 TEM is outfitted with an Aztec X-Max 100 energy-dispersive X-ray spectroscopy (EDX) attachment, allowing for the chemical composition analysis of nanoparticles. Samples for TEM analysis were prepared by depositing a 2 µL droplet of the colloidal solution onto a carbon-coated copper TEM grid, which was then allowed to dry under ambient conditions.

### 2.3. SERS Substrate Fabrication and Measurements

SERS-active substrates were fabricated by depositing 20 µL of the TMDC colloidal solution onto an aluminum substrate. A thin, uniform film was then formed using a spin-coating method. Subsequently, a 2 µL droplet of a crystal violet (CV) dye solution, with concentrations ranging from $10^{-4}$ M to $10^{-10}$ M, was applied to the surface of the TMDC film and allowed to dry for 1 h under ambient conditions. Raman scattering spectra were recorded using a Horiba LabRAM HR Evolution (Horiba, Kyoto, Japan) spectrometer. Measurements were conducted using laser excitation at wavelengths of 532 nm and 633 nm, a 600 grooves/mm diffraction grating, and a 100× microscope objective with a numerical aperture (NA) of 0.9. High spectral reproducibility was confirmed across multiple measurements on each sample.

### 2.4. Enhancement Factor (EF) Calculation

To quantitatively evaluate the performance of the SERS substrates, the enhancement factor (EF) was calculated using the standard formula:

(1) $$EF=\frac{I_{SERS}}{I_{RS}}\times \frac{C_{RS}}{C_{SERS}}$$

where *I_SERS_* and *I_RS_* represent the intensities of a characteristic analyte peak measured on the SERS substrate and in a bulk solution, respectively, under identical spectral conditions. *C_SERS_* and *C_RS_* are the corresponding molar concentrations of the analyte for each measurement. To determine *I_RS_*, a reference normal Raman spectrum was recorded from an aqueous crystal violet solution with a concentration of $10^{-2}$ M (*C_RS_*). This spectrum is provided in the Supporting Information (Figure S1). For each substrate type, SERS spectra were collected at 10 different locations across the substrate surface to account for spatial variability. The intensity of the Raman band at 1620 ${\mathrm{cm}}^{-1}$ was extracted from each spectrum, and the mean intensity was used for *EF* determination. The *EF* values reported in this work, therefore, correspond to calculations based on the mean 1620 ${\mathrm{cm}}^{-1}$ peak intensity, with the corresponding variability.

### 2.5. Photothermal Studies

Photoheating experiments were performed with a tunable titanium–sapphire laser source at a NIR-I wavelength of 830 nm; a collimated laser beam with a spot size ∼2 mm propagates through the colloidal volume (see Supplementary Note S4 for the experimental setup details). The 3.5 mL square quartz cuvette was used with 1 mL of heating colloid. The temperature dynamics were monitored in real time using a calibrated HIKMICRO M10 (Hangzhou Hikvision Digital Technology Co., Ltd., Hangzhou, China) thermal imaging camera. Colloidal extinction was measured using transmission through a cuvette with deionized water as a baseline.

## 3. Results and Discussion

### 3.1. Synthesis Dictates Nanomaterial Morphology While Preserving Crystallinity

Structural analysis via TEM, HRTEM, and SAED (see Figure 1) revealed profound differences in the morphology of the materials produced by the two synthesis techniques, yet, critically, both methods yielded materials that retained their inherent crystalline structure.

As expected, ultrasonic exfoliation, a process involving the mechanical delamination of layered crystals along weakly bonded van der Waals planes, produced large, thin flakes of WSe_2_ and WTe_2_ with irregular shapes. HRTEM analysis and the corresponding SAED patterns confirmed their high degree of crystallinity, showing well-defined atomic planes and sharp, ordered diffraction spots characteristic of the bulk crystal structure. In striking contrast, the femtosecond PLAL method resulted in the formation of quasi-spherical nanoparticles. The most significant finding from this structural characterization is that despite the extreme, non-equilibrium conditions of the PLAL synthesis—involving plasma generation and ultra-rapid quenching—the resulting WSe_2_ and WTe_2_ nanoparticles maintained a high degree of crystallinity. This was unequivocally confirmed by HRTEM images showing clear lattice fringes and SAED patterns exhibiting ordered diffraction rings, analogous to those observed for the exfoliated flakes. This preservation of crystallinity is an important result which effectively eliminates phase transitions and amorphization, as a confounding variable. Complementing the structural analysis, energy-dispersive X-ray spectroscopy (EDX) revealed that while the flakes retained a near-ideal 1:2 stoichiometry, the nanoparticles exhibited chalcogen deficiencies with ratios shifting to approximately 1:1.8 for WSe_2_ and 1:1.7 for WTe_2_ (Table 1). This non-stoichiometry is attributed to the partial evaporation of volatile Se and Te atoms during the high-energy laser ablation process. These findings indicate the presence of chalcogen vacancies in the nanoparticles, contrasting with the chemically pristine exfoliated flakes, and are consistent with our previous report on PLAL-mediated Se vacancies generation in PdSe_2−x_ NPs [47]. Consequently, this allows the investigation to focus exclusively on the effects of morphology and nanoscale structure to explain the observed differences in SERS activity. This establishes a clean and controlled experimental framework for isolating the role of morphology-induced defects in chemical enhancement.

### 3.2. Nanoparticle Morphology Unlocks Superior SERS Performance

Comparative SERS measurements, using crystal violet (CV) as a molecular probe, demonstrated that the substrate morphology is the decisive factor governing its analytical efficacy.

**Figure 2:**
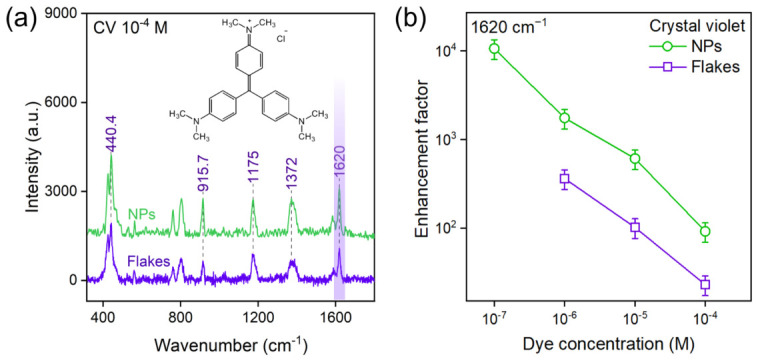
(**a**) Comparison of surface-enhanced Raman scattering (SERS) spectra of $10^{-4}$ M crystal violet (CV) acquired using WSe_2_ flake-based and NP-based substrates; (**b**) enhancement factors (*EF*) calculated for WSe_2_ flake-based and NP-based substrates. Error bars represent the standard deviation.

**Figure 3:**
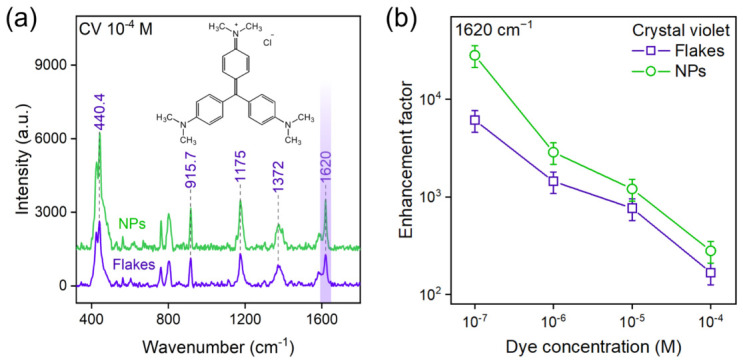
(**a**) Comparison of surface-enhanced Raman scattering (SERS) spectra of $10^{-4}$ M crystal violet (CV) acquired using WTe_2_ flake-based and NP-based substrates; (**b**) enhancement factors (*EF*) calculated for WTe_2_ flake-based and NP-based substrates. Error bars represent the standard deviation.

Figure 2 and Figure 3 present a direct comparison of the Raman spectra and calculated enchancement factors obtained on WSe_2_ and WTe_2_ substrates, respectively. At an analyte concentration of $10^{-4}$ M, the spectra from NP-based and flake-based substrates exhibit intense and clearly resolved characteristic peaks of CV, including the prominent modes at 440, 916, 1175, 1372, 1620 ${\mathrm{cm}}^{-1}$.

The substrates based on WSe_2_ and WTe_2_ nanoparticles exhibited a markedly higher SERS activity compared to their counterparts fabricated from exfoliated flakes. Raman spectra acquired from the nanoparticle-based substrates consistently showed more intense and clearly resolved characteristic peaks of CV, even at low analyte concentrations where signals from the flake-based substrates were weak or undetectable. This observation is fully substantiated by the quantitative analysis summarized in Table 2. The calculated enhancement factors for the NP-based substrates of both WSe_2_ and WTe_2_ were found to be in the range of ∼$10^{4}$.

This represents an enhancement that is consistently an order of magnitude greater than that achieved with substrates made from exfoliated flakes of the same materials, which yielded EFs of approximately $10^{3}$. Furthermore, this enhancement translates directly into improved sensitivity. The limit of detection (LOD) for CV on the nanoparticle substrates reached concentrations as low as $10^{-7}$ M, a full order of magnitude lower than the LOD for the flake-based substrates, which was greater than $10^{-6}$ M.

The EDX-detected chalcogen deficiency, combined with the high-curvature nanoparticle morphology, strongly suggests a higher density of structurally induced active surface sites. We hypothesize that this defect-enriched surface acts as a primary factor facilitating the enhanced SERS activity by creating new channels for resonant photoinduced charge transfer (PICT), while noting that direct atomically resolved quantification of vacancy density is beyond the scope of the present work. The quasi-spherical geometry provides an inherently higher ratio of edge-to-basal-plane area compared to extended flakes. These undercoordinated edge sites are known to be the primary loci for molecular chemisorption and charge exchange in TMDCs. In addition, the high surface curvature of the nanoparticles induces localized strain within the crystal lattice, which promotes a higher intrinsic concentration of point defects, such as chalcogen vacancies. Moreover, the non-equilibrium PLAL synthesis itself, defined by extreme thermodynamic conditions and ultra-rapid quenching rates, kinetically traps a higher density of defects within the nanoparticle structure than is accessible via the quasi-equilibrium exfoliation process. Non-equilibrium synthesis is, therefore, not merely a means of producing small particles, but, rather, a tool for engineering a defect-rich crystalline state. This state constitutes the direct physical origin of the enhanced SERS activity. To construct a physical model for this enhancement, the electronic band alignment between the TMDC substrates and the CV analyte was considered. An efficient PICT-driven chemical mechanism requires a resonance condition, where the excitation laser energy (*E_laser_* = 2.33 eV for 532 nm) facilitates a charge-transfer transition between the analyte’s frontier orbitals and the electronic states of the substrate. While the laser energy is resonant with the HOMO–LUMO gap of the CV molecule (1.9 eV), the substantial SERS enhancement stems from an additional, highly efficient charge-transfer pathway mediated by defect states. Chalcogen vacancies in TMDCs are known to introduce localized electronic states within the material’s band gap. These states act as critical intermediaries, opening new resonant channels for charge transfer—for example, from a TMDC defect level to the LUMO of the excited CV molecule. The high density of such defect states in the PLAL-synthesized nanoparticles—a direct consequence of their abundant edges, high curvature, and non-equilibrium formation—dramatically increases the probability of this resonant charge transfer compared to the near-pristine surfaces of the exfoliated flakes. This amplified charge-transfer efficiency is the fundamental physical origin of the observed order-of-magnitude increase in the enhancement factor.

### 3.3. Colloidal Photothermal Response Governed by Bulk Effective Optical Constants

Achieving the bimodal theranostic capability (SERS + photothermal response) requires a nanomaterial with specific crystalline properties. On the one hand, the chemical mechanism of SERS is understood to be greatly enhanced by a highly disrupted, defect-rich surface. The introduced chalcogen vacancies and undercoordinated edge sites might facilitate a resonant photoinduced charge transfer (PICT) with the adsorbed analyte. On the other hand, to achieve a highly efficient photothermal conversion, especially in case of excitonic TMDCs (WSe_2_) or topological semimetallic TMDCs (WTe_2_), the material should preferably conserve a highly ordered lattice structure of the bulk crystal that supports material-specific electronic band transitions and rapid electron–phonon scattering dynamics. Femtosecond PLAL allows combining the mentioned contradictory properties in a one-step synthesis route. A non-equilibrium PLAL mechanism results in a controlled-defect-engineered nanoparticles with a defect-rich surface (Se and Te vacancies), but remarkably crystalline volume [48,49,50] which is confirmed by HRTEM and SAED analyses (Figure 1d–i).

**Figure 4:**
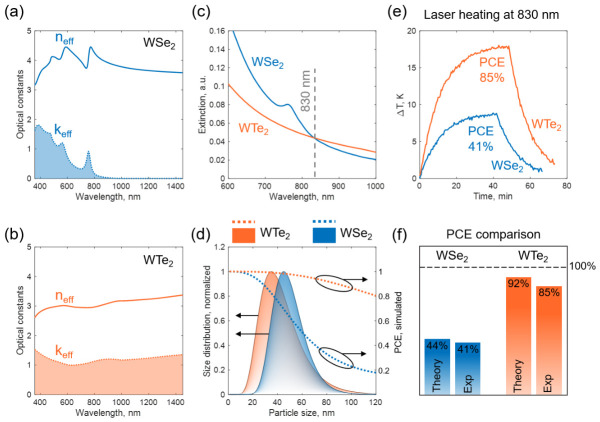
Orientation-averaged effective optical constants of bulk (**a**) WSe_2_ and (**b**) WTe_2_, indicating resonant excitonic optical absorption of WSe_2_ and broadband optical absorption of WTe_2_ in NIR-I. Experimentally measured anisotropic optical constants, used for the orientation-averaged effective constants, were taken from the published works on WSe_2_ [51] and WTe_2_ [52]. (**c**) Measured optical extinction spectra of WSe_2_ and WTe_2_ colloids. (**d**) Dependence of photothermal conversion efficiency on particle size for spherical WSe_2_ and WTe_2_ nanoparticles. (**e**) Experimental photothermal conversion efficiency (PCE) values for femtosecond-laser-ablated WSe_2_ and WTe_2_ nanoparticles at 830 nm heating irradiation. (**f**) Comparison of theoretical and experimental PCE rates of the WSe_2_ and WTe_2_ nanoparticles.

The retention of internal crystallinity is further illustrated by the photothermal analysis. Taking into account the random orientation of colloidal nanoparticles during the laser-induced heating, their optical response is modeled via an effective isotropic medium with an orientation average of the principal components of the dielectric tensor: ${\left(n_{\mathrm{eff}+i}*k_{\mathrm{eff}}\right)}^{2}$ = ϵ_eff_ = 1/3*(ϵ_x_+ϵ_y_+ϵ_z_); optical constants for WSe_2_ were taken from [51] and WTe_2_ from [52]. According to the calculated effective refractive indices of WSe_2_ and WTe_2_ (Figure 4a,b), their optical behavior is dramatically different. The most prominent feature of the WSe_2_ optical spectrum is the pronounced A-exciton transition at approximately 770 nm, which leads to the resonant high optical absorption. However, at the NIR-I photoheating at 830 nm, the WSe_2_ NPs are completely off-resonant and predominantly act as high-refractive (*n_eff_* ≈4) dielectric scatterers.

In contrast, semimetallic WTe_2_ material is characterized by a continuous density of states across the Fermi level, with no energy threshold (bandgap) required to initiate optical transitions. This results in the broadband, highly featureless and high extinction constants (*k_eff_*) shown by the orange curve in Figure 4b. The measured colloidal extinction curves shown in Figure 4c confirm the conservation of WSe_2_ NPs crystallinity via a pronounced A-exciton peak, and a featureless spectrum of WTe_2_ colloid. As the 830 nm excitation is off-resonant, the WSe_2_ extinction decays sharply, indicating that the light–matter interaction is dominated by elastic Rayleigh scattering with only a small absorptive contribution. In contrast, semimetallic WTe_2_ NPs, crystallizing in the distorted orthorhombic Td phase, maintain high extinction values throughout the NIR-I region, suggesting its operation in an absorption-dominated regime. In order to experimentally compare the photothermal response of both colloids, their optical extinctions were aligned at 830 nm, see Figure 4c.

The 830 nm excitation was chosen as a common NIR-I benchmark rather than as the material-specific optimum for either colloid. After matching the total extinction at 830 nm, this comparison probes how each colloid partitions the attenuated optical power into absorption-driven heating versus scattering-dominated losses. Under this fixed-wavelength benchmark, WTe_2_ exhibits a higher photothermal conversion efficiency (PCE) because its semimetallic electronic structure sustains broadband absorption across NIR-I, whereas WSe_2_ operates beyond its principal excitonic absorption maximum and, therefore, retains a larger scattering contribution. A detailed wavelength-dependent PCE study of WSe_2_ colloids, which we performed in [53], reports a maximum PCE value around 60% at the exciton resonance wavelength and the same mean NPs size. Thus, the PCE performance of WSe_2_ is still sufficiently lower than that of semimetallic WTe_2_ colloid (>80%), considered here. A comparison of colloids at the spectral optimum of each material would address a different question, namely, the maximum attainable PCE rather than relative performance under a common operating wavelength.

Preliminary calculations of PCE as a function of NPs size using the effective optical constants from Figure 4a,b for the 830 nm heating wavelength reveal much higher PCE values for WTe_2_ NPs with respect to WSe_2_ NPs, for a wide range of particle sizes from near-zero to >100 nm (see Figure 4d). The photothermal response generally increases for smaller particles. Therefore, additionally taking into account a slightly smaller mean size of WTe_2_ colloidal particles (∼35 nm) than WSe_2_ colloidal particles (∼45 nm), the WTe_2_ colloid should be much more effective in the photoheating. Macroscopic photoheating experiments, performed by irradiating the quartz cuvettes filled with colloids, approve the theoretical predictions. In both systems, the temperature–time profiles exhibit three characteristic stages (see Figure 4e): (i) a rapid initial rise during the first 15–20 min, where photothermal heat generation by the NPs exceeds heat loss to the surroundings; (ii) a plateau steady-state region (in 20 min after the irradiation start), in which the laser-induced heat input is balanced by dissipation through the cuvette walls; and (iii) an exponential cooling phase upon laser shutdown (in ≈40 min after the irradiation start), governed solely by the thermal relaxation of the system. The maximum steady-state temperature increase (Δ*T_max_*) is Δ*T_max_*≈8.5 K for WSe_2_ and ≈18 K for WTe_2_ under identical experimental conditions. Taking into account the experimental colloidal polydispersity, the size-averaged PCE values were theoretically calculated for both colloids (see Figure 4f), by using the method described in [54,55] (see Supporting Information, Supplementary Notes S2 and S3). The thermograms fitting parameters within the used model are shown in Supplementary Table S1; in particular, steady-state temperature increases $\xi_{WSe2}=8.98$ K and $\xi_{WTe2}=18.11$ K are well correlated with the direct data from Figure 4e. The heat loss coefficients $\tau_{WSe2}=693$ s and $\tau_{WTe2}=670$ s are almost identical, which reflects the similar environmental conditions during the PCE experiments. In the similar environment, with aligned optical extinctions and the same heating power, the steady-state temperature is proportional to the PCE value, which is, indeed, demonstrated here. The small but systematic reduction in the experimental PCEs relative to their theoretical counterparts is attributed to morphological factors not captured by the idealized Mie model, for example, a minor aggregation of nanoparticles resulting in their higher effective size and, consequently, lower effective PCE. Nevertheless, theoretical predictions derived from Mie theory using orientation-averaged bulk optical constants demonstrate strong agreement with the experimental data, yielding a relative error of less than 10%.

### 3.4. Benchmarking and Suitability for Biological Environments

When benchmarked against representative chemically enhancing SERS platforms, purely pristine graphene or conventional 2H-MoS_2_ flakes generally plateau at *EFs* of $10^{1}$–$10^{3}$ [22,56,57]. Attaining *EFs* of ∼$10^{4}$ without plasmonics highlights the efficacy of the PLAL-induced active sites. While metastable phase-engineered materials (e.g., 1T-MoS_2_ [58]) can reach similar or higher *EFs*, they suffer from rapid ambient oxidation, whereas our PLAL-generated nanoparticles ensure robust environmental stability via their preserved crystalline cores.

For theranostic applications, biocompatibility is paramount. A transformative advantage of the femtosecond PLAL technique is its intrinsically “green” nature [40]. Synthesized in pure deionized water, the resulting nanoparticles are completely ligand-free, lacking the toxic organic solvents or persistent surfactants (e.g., CTAB) common in chemical synthesis. Additionally, recent studies show that WSe_2_-based nanosystems have already been explored in combined therapeutic settings and that tungsten-based TMDCs can exhibit a favorable physiological degradation pathway, gradually oxidizing into highly water-soluble tungstate (${\mathrm{WO}}_{4}^{2-}$) species that facilitate safe renal clearance [59,60]. In this context, it is also important to note that WTe_2_ is comparatively surface-sensitive and may require passivation or appropriate functionalization to ensure colloidal/chemical stability in biologically relevant environments [61,62]. Collectively, these considerations establish a robust physicochemical proof-of-concept toward dual-mode theranostic development.

## 4. Conclusions

This study provides a systematic comparison of the plasmon-free SERS activity and photothermal capabilities of crystalline TMDCs, unequivocally demonstrating that morphology is a critical parameter for governing both chemical enhancement and optical response. The central finding of this work is that crystalline nanoparticles of WSe_2_ and WTe_2_, synthesized via non-equilibrium femtosecond laser ablation (PLAL), consistently exhibit superior SERS performance (*EF*∼$10^{4}$) compared to their highly ordered, flake-like analogues produced by conventional exfoliation (*EF*∼$10^{3}$).

This pronounced SERS enhancement exhibits a strong correlation with the significantly higher density of structurally induced active sites—including edge sites, point defects, and high-curvature regions—that are inherent to the nanoparticle morphology. A defect-mediated charge transfer model explains this superiority: the dense landscape of active sites creates a high concentration of electronic states within the TMDC band gap, acting as resonant intermediaries that facilitate efficient photoinduced charge transfer with the analyte molecule.

Crucially, this work demonstrates that the PLAL synthesis successfully resolves the conflicting material requirements for theranostic applications. While the technique induces the necessary surface defects for SERS, it simultaneously preserves the high-quality crystalline lattice of the nanoparticle volume. This structural conservation allows for the accurate prediction of optical behaviors using bulk dielectric constants and enables efficient photothermal performance. Specifically, we confirmed that the semimetallic character of WTe_2_ is retained in the nanoparticle form, resulting in a broadband absorption-dominated regime and a superior photothermal conversion efficiency (>80%) in the NIR-I window, significantly outperforming the scattering-dominated response of excitonic WSe_2_ (>40%).

Finally, this research establishes morphology engineering via non-equilibrium synthesis as a robust and generalizable design strategy.

## Figures and Tables

**Table 1:** Atomic composition and stoichiometry of WSe_2_ and WTe_2_ materials determined by energy-dispersive X-ray spectroscopy (EDX).

Materials		Atomic Composition (at.%)		Stoichiometry
		**W**	**Se/Te**	
WSe_2_	Flakes	30.51	61.77	∼1:2
	NPs	31.08	56.31	∼1:1.8
WTe_2_	Flakes	29.54	59.15	∼1:2
	NPs	26.20	44.38	∼1:1.7

**Table 2:** Comparative surface-enhanced Raman scattering (SERS) performance of crystalline WSe_2_ and WTe_2_ substrates.

Substrate	Synthesis Method	Morphology	Analyte	EF	LOD
WSe_2_	Ultrasonic Exfoliation	Crystalline Flakes	CV	3.6 ± 0.9 × $10^{3}$	$10^{-6}$ M
WSe_2_	Femtosecond PLAL	Crystalline Nanoparticles	CV	1.1 ± 0.3 × $10^{4}$	$10^{-7}$ M
WTe_2_	Ultrasonic Exfoliation	Crystalline Flakes	CV	6.1 ± 1.5 × $10^{3}$	$10^{-7}$ M
WTe_2_	Femtosecond PLAL	Crystalline Nanoparticles	CV	2.8 ± 0.7 × $10^{4}$	$10^{-7}$ M

## Data Availability

All data generated or analyzed during this study are included in this published article.

## Supplementary Materials

The following supporting information can be downloaded at: https://www.mdpi.com/article/10.3390/nano16090546/s1.

## Abbreviations

The following abbreviations are used in this manuscript:

LSPR	Localized surface plasmon resonances
CM	Chemical mechanism
PICT	Photoinduced charge transfer
DI water	Deionized water
TMDC	Transition metal dichalcogenide
NP	Nanoparticle
PLAL	Pulsed laser ablation in liquid
TEM	Transmission electron microscope
HRTEM	High-resolution transmission electron microscope
SAED	Selected area electron diffraction
EDX	Energy-dispersive X-ray spectroscopy
SERS	Surface-enhanced Raman scattering
CV	Crystal violet
LOD	The limit of detection
EF	Enhancement factor
NA	Numeric aperture
PCE	Photothermal conversion efficiency
