# Supplementary material for: Morphology-Driven SERS Activation in TMDCs: A Dual-Mode Platform for Sensorics and Theranostics
Source: Nanomaterials (Basel). 2026 Apr 30;16(9):546. doi: 10.3390/nano16090546 (PMC13165396; doi:10.3390/nano16090546)
Supplement: Supplementary file 1 [file nanomaterials-16-00546-s001.zip › nanomaterials-4252612-supplementary.pdf]

Supporting Information for

Morphology-Driven SERS Activation in TMDCs: A Dual-Mode Platform for Sensorics and Theranostics

N.M. Belozerova[1,2], A.A. Ushkov[1], D.V. Dyubo[1], A.V. Syuy[1,3,4], A.I. Chernov[1], A.A. Vyshnevyy[1,3], S.M. Novikov[1], G.I. Tselikov[3], A.V. Arsenin[1,3], V.G. Leiman[1] and V.S. Volkov[3*]

[1]Moscow Center for Advanced Studies, Kulakova Str. 20, Moscow, 123592, Russia
[2]Frank Frank Laboratory of Neutron Physics, Joint Institute for Nuclear Research, Joliot-Curie 6, Dubna, 141980, Russia
[3]Emerging Technologies Research Center, XPANCEO, Internet City, Emmay Tower, Dubai, United Arab Emirates
[4]Department of General Physics, Perm National Research Polytechnic University, Perm, 614990, Russia
[*]Correspondence: vsv@xpanceo.com

Supplementary Note S1.

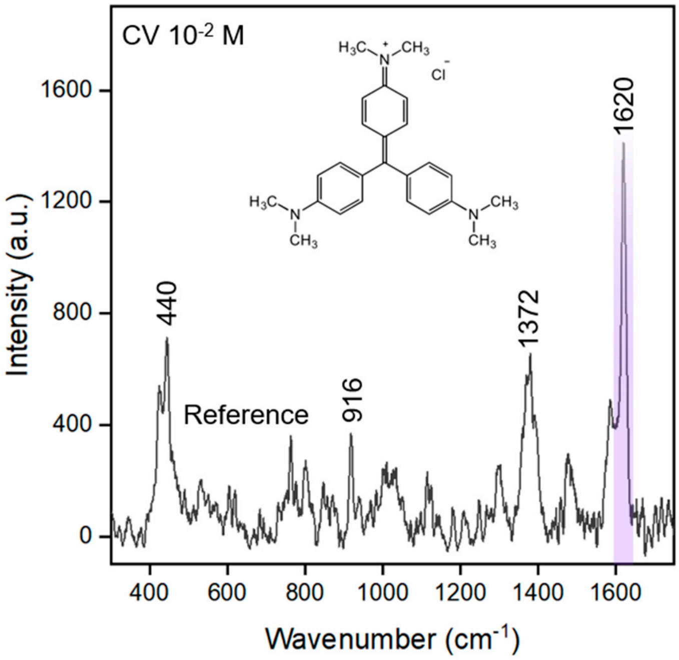

Supplementary Figure S1. Reference normal Raman spectrum of crystal violet (CV) used to calculate the enhancement factor (EF). The spectrum was recorded from an aqueous CV solution at a concentration of 10^{-2} M. The intensity of the band at 1620 cm^{-1} (marked) was used as I_{ref}.

Supplementary Note S2.

To calculate the photothermal conversion efficiency η we follow an approach described in [S1]. For relatively low heatings the master equation for the thermogram *ΔT(t)* can be written as:

$$C \cdot \dot{\Delta T} = P_{abs} - L \cdot \Delta T(t), \quad \text{(Eq. S1)}$$

where C is the heat capacity ($C \cong m_{water} C_{water}$), L is the linear losses coefficient, P_{abs} is the optical power absorbed by NPs, dot above the variable denotes the time derivative. In photoheating experiments we used 1 ml water colloids of NPs.

Under the laser irradiation P_{abs}>0 the solution of Eq. S1 is a "heating curve":

$$\Delta T(t) = \xi + B \cdot \exp(-t/\tau), \qquad \text{(Eq. S2)}$$

where $\xi = P_{abs}/L$, $\tau = C/L$, ξ is defined from initial conditions.

In the absence of laser irradiation (P_{abs}=0) the solution of Eq. S1 is a "cooling curve":

$$\Delta T(t) = \Delta T_{max} \cdot \exp\left(-\frac{t}{\tau}\right), \qquad \text{(Eq. S3)}$$

where ΔT_{max} is the colloid temperature at the moment when the laser was turned off, see Supplementary Figure S2a.

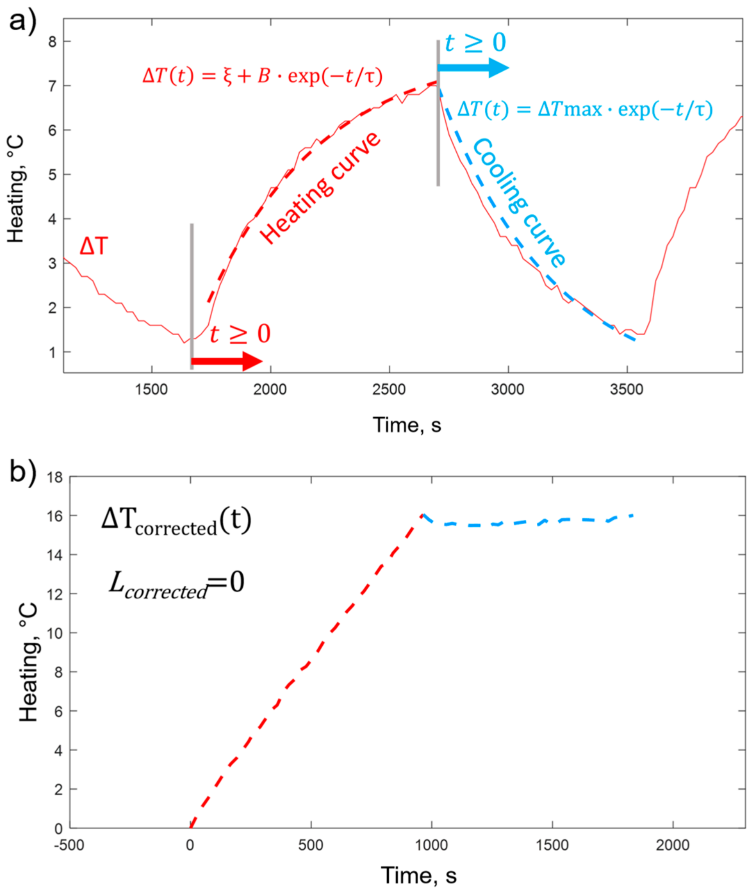

Supplementary Figure S2. a) Definitions of "heating" and "cooling" curves and their fitting using Eqs. S2,S3; b) The same thermogram as in a), re-calculated in the adiabatic representation via Eq. S4.

After some math it is possible to prove that the following change of coordinates:

$$\Delta T_{corrected}(t) = \int \left(\dot{\Delta T} + \frac{\Delta T}{\tau}\right) dt \qquad \text{(Eq. S4)}$$

allows to exclude the losses coefficient L from the system, thus making it adiabatic ($L_{corrected}$=0):

$$C \cdot \dot{\Delta T}_{corrected} = P_{abs}, \qquad \text{(Eq. S5)}$$

see Supplementary Figure S2b.

In adiabatic representation, the photothermal conversion efficiency η can be obtained as

$$\eta = C\frac{\dot{\Delta T}_{corrected} - \dot{\Delta T}_{corrected\ water}}{P_0(1 - Tr)} \qquad \text{(Eq. S6)}$$

where $\dot{\Delta T}_{corrected\ water}$ is calculated for the pure water, P_0 is the laser beam power after it passes the pure water cuvette and $P_0 \cdot Tr$ is the power after the actual water colloid of NPs.

Taking into account that P_{abs} is

$$P_{abs} = C \cdot \left(\dot{\Delta T}_{corrected} - \dot{\Delta T}_{corrected\ water}\right), \qquad \text{(Eq. S7)}$$

colloidal extinction is

$$I_{ext} = 1 - Tr \qquad \text{(Eq. S8)}$$

colloidal absorption is

$$I_{abs} = P_{abs}/P_0 \qquad \text{(Eq. S9)}$$

the colloidal absorption curve in Fig.4f can be obtained from the photoheating experiment as

$$I_{abs} = \eta \cdot I_{ext} \qquad \text{(Eq. S10)}$$

Supplementary Note S3.

Photothermal conversion efficiency (PCE, η) of colloidal solutions was modeled by using NPs extinction and absorption cross sections, obtained from Mie theory. During the propagation in the colloid the incident heating laser beam is being scattered and absorbed by nanoparticles. The PCE value, compatible with experimental value considered in Supplementary Note S2, is defined as:

$$\eta = P_{abs}/P_{ext}, \qquad \text{(Eq. S11)}$$

where P_{abs} is the total power, absorbed by the NPs, and P_{ext} is the total power, absorbed and scattered by the NPs.

The absorption (c_{abs}) and extinction (c_{ext}) cross sections of NPs are defined as [S2]:

$$c_{abs} = P_{abs}/I_0 \qquad \text{(Eq. S12)}$$

$$c_{ext} = P_{ext}/I_0, \qquad \text{(Eq. S13)}$$

where I_0 is the intensity of a wave incident on NP.

The laser beam with initial power P_0 is attenuated after propagation a distance *x* in a colloid:

$$P(x) = P_0 \exp\left(-nxc_{ext}\right), \qquad \text{(Eq. S14)}$$

where *n* is NPs volume concentration.

Consequently, the total power, removed from the optical beam is:

$$P_{ext}(x) = P_0[1 - \exp\left(-nxc_{ext}\right)], \qquad \text{(Eq. S15)}$$

The total optical power P_{abs} absorbed by NPs after propagation a distance *x* in a colloid:

$$P_{abs}(x) = P_0[1 - \exp\left(-nxc_{ext}\right)] \cdot c_{abs}/c_{ext} \qquad \text{(Eq. S16)}$$

Thus, substituting Eqs. S15 and S16 into Eq. S11 we get a theoretically expected value of PCE:

$$\eta = c_{abs}/c_{ext}, \quad \text{(Eq. S17)}$$

which is valid for low-concentrated (to prevent interparticle optical interactions) and monodisperse colloids. In many cases, Eq. S11 correctly estimates PCE value even for polydisperse systems, where the size distribution is not too broad (as, for example, in Fig.2c of the main text).

It can be shown that in case of polydisperse colloids Eq. S11 should be slightly modified: optical cross-sections should be replaced with effective ones, averaged by using statistical size distributions of nanoparticles in a real colloid.

In our study we use a lognormal distribution with the following probability density function:

$$f(D) = \frac{1}{D\sigma\sqrt{2\pi}} \exp\left(-\frac{(\mathrm{lnD}-\mu)^2}{2\sigma^2}\right), \quad \text{(Eq. S18)}$$

where μ and σ are fitting parameters obtained from experimental data (see Fig.2c of the main text). The distribution mode (the value with the highest probability) and variance are defined as follows:

$$mode = \exp\,(\mu - \sigma^2) \quad \text{(Eq. S19)}$$

$$variance = [\exp(\sigma^2) - 1]\exp\,(2\mu + \sigma^2) \quad \text{(Eq. S19)}$$

References

S1. Abu Serea, Esraa Samy, et al. "Enhancement and tunability of plasmonic-magnetic hyperthermia through shape and size control of Au: fe3O4 janus nanoparticles." ACS Applied Nano Materials 6.19 (2023): 18466-18479.

S2. Hohenester, Ulrich. Nano and quantum optics: an introduction to basic principles and theory. Springer Nature, 2019.
